# Shikonin Functionalized Packaging Film for Monitoring the Freshness of Shrimp

**DOI:** 10.3390/ma15196615

**Published:** 2022-09-23

**Authors:** Swarup Roy, Parya Ezati, Deblina Biswas, Jong-Whan Rhim

**Affiliations:** 1 Department of Food and Nutrition, BioNanocomposite Research Institute, Kyung Hee University, 26 Kyungheedae-ro, Dongdaemun-gu, Seoul 02447, Korea; 2 School of Bioengineering and Food Technology, Shoolini University, Bajhol 173229, India

**Keywords:** shikonin, gelatin, cellulose nanofibers, smart packaging, active packaging, antibacterial activity, food freshness

## Abstract

A shikonin embedded smart and active food packaging film was produced using a binary mixture of gelatin and cellulose nanofiber (CNF). Shikonin is an alcohol-soluble natural pigment extracted from *Lithospermum erythrorhizon* root. The fabricated film showed good pH-responsive color changes and volatile gas sensing properties. Moreover, the film exhibited excellent antioxidant and antibacterial activity against foodborne pathogens. The shikonin incorporated gelatin/CNF-based film showed excellent UV-light barrier properties (>95%) and high tensile strength (>80 MPa), which is useful for food packaging. The hydrodynamic properties of the film were also slightly changed in the presence of shikonin, but the thermal stability and water vapor permeability remained unaffected. Thus, the inclusion of shikonin in the gelatin/CNF-based film improves not only the physical properties but also the functional properties. The film’s color indicator properties also clearly show shrimp’s freshness and spoilage during storage for 48 h. The shikonin-based functional film is expected to be a promising tool for multi-purpose smart and active food packaging applications.

## 1. Introduction

Currently, active and intelligent packaging is a trending tool to handle food spoilage and preservation issues. Active packaging is useful to enhance the shelf-life of food products, whereas intelligent packaging can sense and monitor the quality of the food during storage [1,2]. In intelligent packaging, the colorimetric process is the simplest, and it can perceive visual changes in food during spoilage in real-time [3,4]. The food quality changes during storage are subjected to the formation of ethylene, carbon dioxide, and other volatile gases, as well as microbial attacks and oxidation [5,6]. There is an ample number of chemicals available in the market, such as ethylene scavengers (potassium permanganate, titanium dioxide, etc.), oxygen scavengers (gallic acid, iron, palladium, etc.), carbon dioxide emitters (ferrous carbonate, sodium bicarbonate, etc.), and preservatives (potassium sorbate, sodium benzoate, sodium nitrite, etc.) to protect foods from deterioration [7,8]. Excessive use of chemicals in foods creates a serious concern about food safety and security. In this regard, natural color indicator included intelligent pH responsive volatile gas sensing and detection can be advantageous to monitor food quality in real-time. Recently, many reports have been published regarding intelligent color indicator film for food freshness indicator using anthocyanin as a natural color indicator [9,10,11]. Curcumin has also received much use as a bioactive, natural, intelligent, and active packaging materials [2,12].

Generally, in smart packaging, a color indicator is used and to this end a natural colorant is superior to a synthetic dye due to toxicity and other hazards [13,14]. In natural dye, anthocyanin is mostly used so far, but recently shikonin has received much interest from researchers due to its excellent color stability and good color-changing potential [14,15]. Shikonin can be easily extracted from the root of gromwell (*Lithospermum erythrorhizon*), and it showed good color variation in acidic and alkaline conditions due to the structural transformation [16,17]. Shikonin is the primary red color bioactive pigment in the gromwell herb and has been used as a medicinal herb since ancient days [18]. Shikonin is also known to show excellent antioxidant and antibacterial activity; as a result, it can also be used as an active packaging ingredient [19,20,21]. Thus, natural plant-derived color shikonin is an ideal candidate for active and smart food packaging applications as a bioactive ingredient.

The packaging film made from bio-based natural sources is beneficial in terms of an environmental prospect over petroleum-derived films [22]. Gelatin is an animal-derived protein-based biopolymer commonly used in food packaging systems due to its excellent film-forming potential [23,24]. The physical properties of the gelatin-base can be improved by blending it with other polymers. To this end, cellulose nanofiber can be a good option due to its fibrous character and high rigidity [25,26]. Cellulose nanofiber (CNF), a natural polymer derived from cellulose, contains many hydroxyl groups, making it suitable for mixing with gelatin [27,28]. The gelatin and cellulose nanofiber (CNF) can interact via crosslinking and chemical bonding; thus, the blended film is expected to show better physical performance [28,29]. Very recently, a few shikonin-based color indicator films have been reported as using various types of polymer matrix [15,17,30,31,32,33], but so far no report is available regarding the gelatin/CNF/shikonin-based colorimetric packaging system.

This work aims to fabricate pH-dependent color-changing indicator film using shikonin and gelatin/CNF. The pH and volatile gas sensitivity were determined. The film’s optical, thermal, hydrodynamic, mechanical, antioxidant, and antimicrobial properties were evaluated, and the use of the film on shrimp preservation and freshness monitoring was studied.

## 2. Materials and Methods

### 2.1. Materials

Food grade gelatin (Type A, 200 Bloom) was acquired from Gel-Tec Co., Ltd. (Seoul, Korea). CNF (CNF^TEMPO^, W: 10–20 nm, L: 3–350 µm, density: 1.0 g/cm^3^) was obtained from Moorim P&P Co., Ltd. (Ulsan, Korea). Glycerol was obtained from Daejung Chemicals & Metals Co., Ltd. (Siheung, Gyeonggi-do, Korea). Shikonin was extracted from the root of *Lithospermum erythrorhizon* using a previously reported method [17]. All other chemicals used were analytical reagent grade.

### 2.2. Fabrication of Films

A total of 4 g of gelatin and 100 mL of the aqueous CNF suspension (1 wt%) were first mixed and then 30 wt% glycerol was added as a plasticizer to this mixture, heated at 70 °C for 25 min with constant stirring to produce completely soluble film-forming polymer solution [28]. The film-making solution was cooled to 40 °C and then 10 wt% of shikonin based on polymers (*w*/*w*) was mixed and stirred for half an hour. The film-forming solution was then cast on a flat Teflon film-coated glass plate (0.24 m × 0.3 m) and dried at room temperature (22 ± 3 °C) for 48 h. The dried film was peeled off the plate and conditioned (25 °C and 50% RH). The fabricated film samples were designated as Gel/CNF, Gel/CNF/ShK, depending on the composition. Detailed methods for the film’s characterization are shown in the Supporting Information.

## 3. Results and Discussion

### 3.1. Properties of the Film

#### 3.1.1. pH-Dependent Color Change of the Films

**Figure 1:**
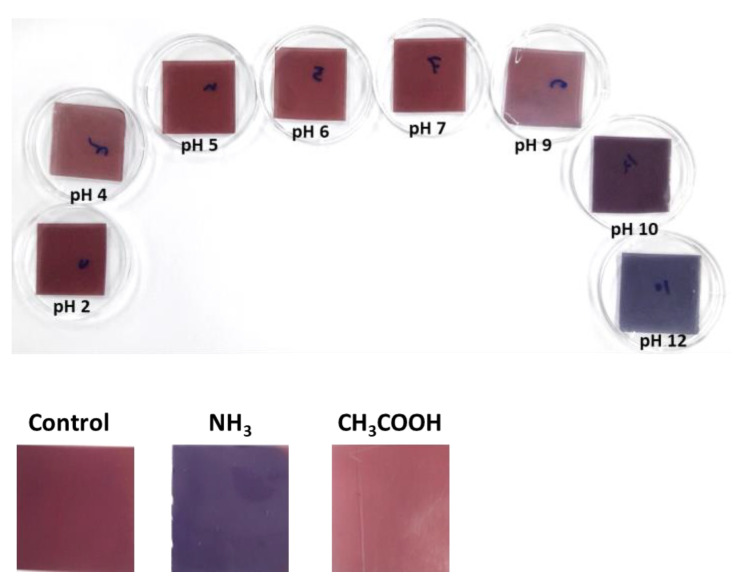
pH-responsive color change of the indicator film and volatile gas sensitivity of the film.

The color response of the fabricated film at various pHs from 2 to 12 is presented in Figure 1, and the color values are shown in Table S1 (Supplementary Materials). When pH changed from 2 to 12, the film’s color changed sharply from reddish to blue. The film’s pH-dependent color variations follow a similar pattern to the color changes of the shikonin itself, as shown in Figure S1 (Supplementary Materials). The color variation is presumably due to the structural alteration of shikonin in the presence of acidic and basic environments. The observed finding is consistent with the formerly reported shikonin added color indicator film [15,17]. The increase in pH generally reduces the *a*- and *b*-values, although the changes were inconsistent in the acidic pH. The inconsistent color variation of film in the acidic pH was due to the color stability of shikonin in <pH 7; as a result, it remained reddish. The pH-responsive color variation of the film is analogous to the previously reported shikonin-based color indicator [19,31]. In addition, the change in L-value and the overall color difference were also inconsistent, and a slightly irregular pattern was observed in the acid-base environment. Compared with the color change of only shikonin solution, the color indicator film’s color was not changed sharply, which could be due to the difference in the diffusion rate of hydrogen ions under liquid and solid media [30,34]. Moreover, the film matrices have also impacted the indicator’s color change variation.

The color sensitivity of the film to volatile gas is also presented in Figure 1. When the film is exposed to volatile ammonia, its color changes rapidly from red to blue, and when it comes into contact with volatile acetic acid, it becomes bright red. These results indicate that the film efficiently monitors food freshness in real time. Similar results were reported earlier in the case of shikonin, including gelatin/carrageenan and CMC/agar-based smart indicator films [14,33].

#### 3.1.2. Surface Color and Optical Properties

The surface color of the films is illustrated in Table 1. The control film showed a high lightness (L-value) but was reduced to half in the presence of a color indicator, owing to the shikonin’s red color. As anticipated, the a-value sharply changed from −0.7 to 31 due to the incorporation of shikonin. Interestingly, the presence of shikonin did not alter the b-value of the film. The film’s total color difference was also significantly increased due to the addition of shikonin. The detected results are comparable with the earlier reported shikonin added to different biopolymer-based films [17,19,31]. The whiteness (WI) of the film showed a sharp decrease (~25%) compared to the control film due to the dark red color of shikonin.

**Figure 2:**
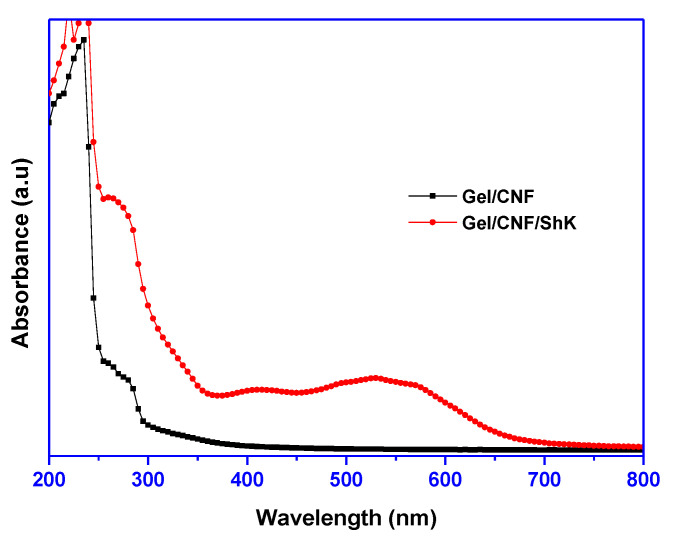
UV-vis absorption spectra of the smart films.

The UV-vis absorption spectra of the fabricated film are presented in Figure 2. Shikonin was added as a natural colorant in the color indicator films, and its presence can be perceived from the absorption spectra. In the case of shikonin-added film, an absorption peak was detected at approximately 550 nm due to the existence of shikonin in the film matrix [30,34]. Shikonin visible light absorption chemistry is a well-established phenomenon [18]. The formation of the identical peak in the film confirms the presence of shikonin without any significant modification during the formation of the films. Analogous absorption profiles of shikonin in various types of other bio-based polymers have been detected in a formerly published report [17,19,31].

The UV-light barrier properties and transparency results of the films are also presented in Table 1. It can be observed that the neat film presented some UV-light barrier properties that originated from gelatin [35]. Nevertheless, the UV-light barrier properties are highly enhanced due to the blending of shikonin in the polymer matrices. The increase in UV-light barrier properties was ~94%, indicating very strong UV-light absorption properties, and it is advantageous to preserve UV-light sensitive packaging. The pristine film was highly transparent, but the presence of shikonin significantly decreased it from ~90 to 70%, but still high enough for food packaging application. The obtained transparency and UV-light barrier properties of the shikonin-based film are similar to the formerly reported data [13,17].

#### 3.1.3. Microstructure

**Figure 3:**
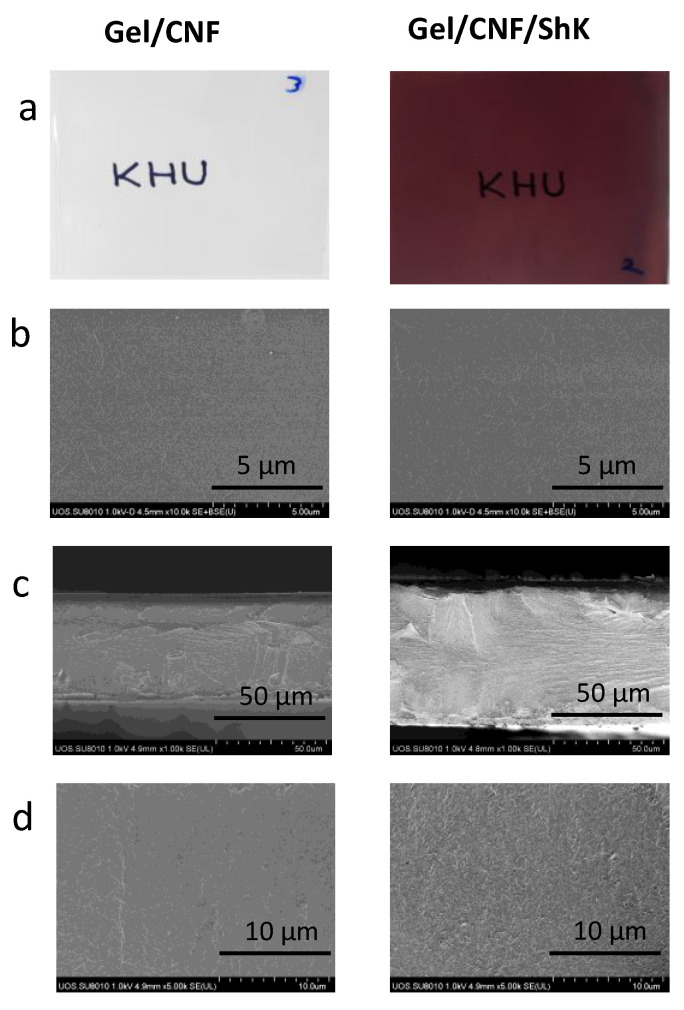
Apparent image and morphology of the smart films. (**a**) apparent image, (**b**) surface SEM image, (**c**,**d**) cross-sectional SEM images.

The visible form of developed films and microstructure is shown in Figure 3. As expected from the optical properties, the neat film is colorless, while the shikonin included film is reddish. From the microscopic analysis, the neat film is compact and compatible, mainly owing to the crosslinking interaction among the polymers (gelatin and cellulose) [27]. Both neat film and shikonin-added films are intact without any pores. The association of shikonin in the Gel/CNF matrices did not alter the apparent surface morphology in the film. 

Shikonin is well dispersed in the polymer matrix and shows good compatibility. The cross-section morphology of the film also presented good dispersion of shikonin, but the roughness of the shikonin-added film seems to be increased, which is most likely due to the presence of shikonin in gelatin/cellulose matrices. A similar microstructural morphology was observed in the case of shikonin-added gelatin/carrageenan-based film in a previously published report [19].

#### 3.1.4. FTIR 

**Figure 4:**
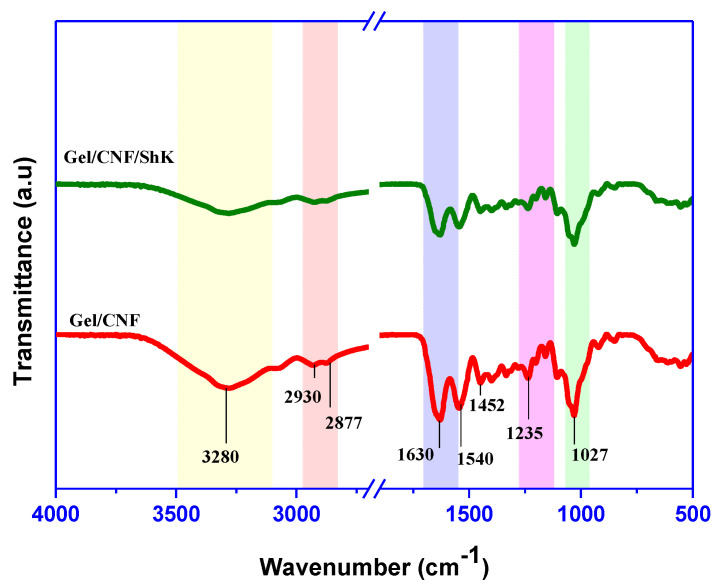
FTIR spectra of the smart films.

FTIR spectra of the developed film are shown in Figure 4. The chemical interaction between shikonin and gelatin/cellulose polymer is depicted in FTIR. The primary chemical groups detected in the FTIR spectra are 3281 (-OH), 2932 & 2875 (-C-H), 1632 (-CO-NH), 1541 (-N-H), 1455 (-C-N), 1233 (-N-H), and 1028 (-C-O-C) cm^−1^ [36,37,38]. Moreover, peaks for both gelatin (amide (1540, 1235 cm^−1^), peptide (1630 cm^−1^)), and cellulose structure (pyranose (1027 cm^−1^)) are detected [28], which suggests the proper blending of the two types of polymers. The association of shikonin has not induced a new peak or a striking chemical shift in peak position; nevertheless, a slight alteration in some peaks was detected (3281, 1632, 1028 cm^−1^, etc.). The observed slight alteration in the FTIR peaks most likely comes from the H-bonding and other physical interactions [28]. A comparable result was detected earlier in the case of shikonin blended cellulose/agar-based color indicator film [39].

#### 3.1.5. Thermal Stability

**Figure 5:**
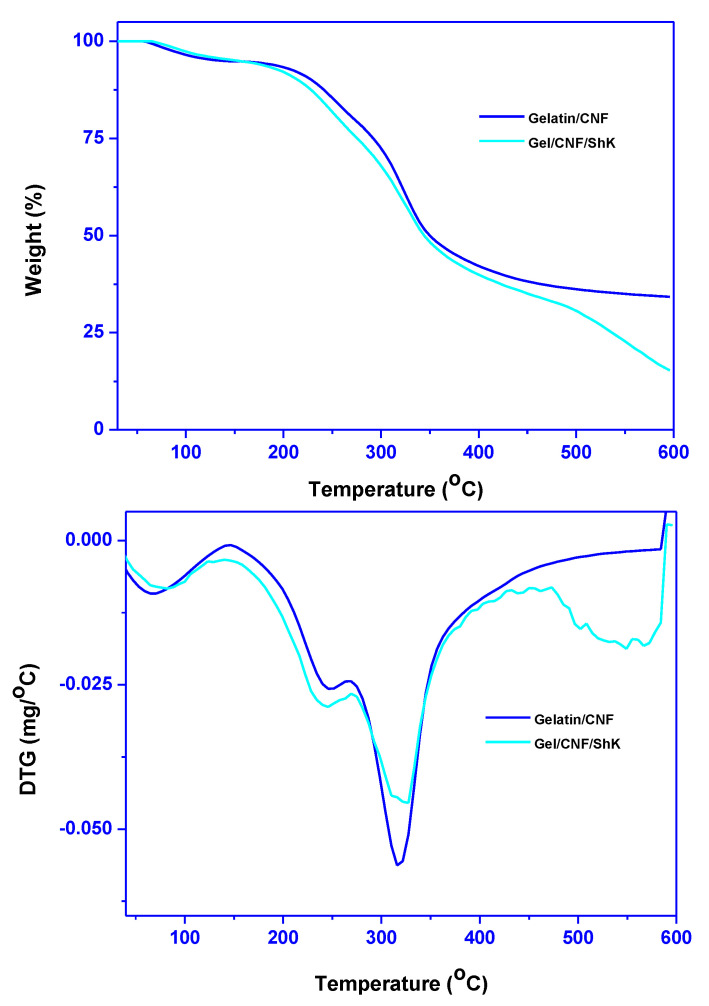
TGA and DTG thermograms of the smart film.

The thermal stability of the film (TGA and DTG) is shown in Figure 5. This study is needed to understand the thermal behavior of the product and packaging material. Multi-step weight loss was detected in the thermal analysis. The initial maximum weight loss was observed at approximately ~70 °C due to the evaporation of water vapor [40]. Second maximum weight loss was spotted at ~220 °C caused by the degradation of glycerol and low molecular weight protein [40,41]. The third maximum weight loss was perceived as ~300 °C corresponds to the decomposition of cellulose and high molecular weight proteins of gelatin [42]. When shikonin was added to the gelatin/cellulose-based film, the weight loss pattern was similar, but in the first stage, weight loss was slightly reduced, while in the second stage, weight loss increased, whereas in the third case, it again decreased. Previous reports on the thermal degradation pattern of shikonin added carboxymethyl cellulose/agar-based film and gelatin/carrageenan-based were well agreed with the current findings [14,19]. These results indicate the possibility of interaction between shikonin and the polymer matrices. As a whole, the thermal stability of the film was not much altered in the presence of shikonin. Interestingly, the final char decreased sharply in the case of shikonin-added film (34% to 15%) compared to its counterpart, which could be due to the polyphenols present in shikonin. 

#### 3.1.6. Mechanical Properties 

To ensure that the packaging film can withstand external stress and keep its integrity during its use as food packaging, it is imperative to improve its mechanical qualities [43]. Table 2 shows the thickness and mechanical characteristics of the films. Shikonin significantly changed the mechanical characteristics of Gel/CNF film. In general, the distribution and density of components, the type and concentration of additives, and the intra- and intermolecular interactions amongst polymer matrices all significantly determine the mechanical properties of composite films [44]. The addition of shikonin did not significantly change (*p* > 0.05) the thickness of the Gel/CNF/ShK composite film. This may be because shikonin was evenly distributed throughout the Gel/CNF polymer matrix, creating a compatible composite, as shown by the SEM data (Figure 3). The inclusion of shikonin improved the Gel/CNF film’s tensile strength (TS), elongation at break (EB), and elastic modulus (EM). Shikonin may have been added in the proper quantity, which resulted in the intermolecular interactions that formed a compatible complex between its polyhydroxy groups and the hydrophilic groups of the Gel/CNF and improved the film’s tensile strength [44]. 

#### 3.1.7. Hydrodynamic Properties

The hydrodynamic properties of the films, such as water vapor permeability (WVP) and water contact angle (WCA), moisture content (MC), water solubility (WS), and swelling ratio (SR), are presented in Table S2 (Supporting Information). The shelf life of food is affected by the water vapor transmission rate of the packing material. The film should have a low water vapor transmission rate [45,46,47]. Shikonin’s inclusion slightly raised the water vapor pressure (WVP) of the Gel/CNF film. This increase in WVP may be attributable to shikonin’s hydrophobicity in the polymer matrix, which partially breaks up the film’s continuous structure and increases water vapor flow through the film [48]. The WVP of biopolymer-based films has frequently risen with the addition of various fillers, which is consistent with the current finding [49].

The hydrophobicity of packing films can be measured using the water contact angle value; the higher the angle, the more hydrophobicity there will be [50]. An increase in contact angle was observed with the addition of shikonin, and the Gel/CNF/ShK film showed an increase from 60.3 ± 1.0° to 66.5 ± 3.4°. The increase in contact angle may have resulted from the shikonin’s hydrophobicity [51]. The findings of the contact angle match the rate of water vapor transmission.

Films with antibacterial/antioxidant properties must be water-resistant to preserve food with intermediate or high moisture levels. The addition of the shikonin did not greatly improve the film’s MC, which was 6.7%. Gel/CNF film had a lower WS than Gel/CNF/ShK film, but the difference was not statistically significant (*p* > 0.05). Shikonin’s addition increased the amount of hydrophobic functional groups, which further contributed to the films’ low solubility compared to shikonin-free Gel/CNF film. According to Roy et al. (2021), an increase in hydrophobic functional groups due to the addition of shikonin reduced the decomposition and disintegration of cellulose nanofiber films [17]. The addition of shikonin showed a significant decrease in the swelling ratio of the film, which was in good agreement with reported studies [19]. The considerable decrease in the composite film’s swelling ratio may be attributable to the addition of shikonin, which increased the hydrophobicity of the films.

### 3.2. Antimicrobial Activity

**Figure 6:**
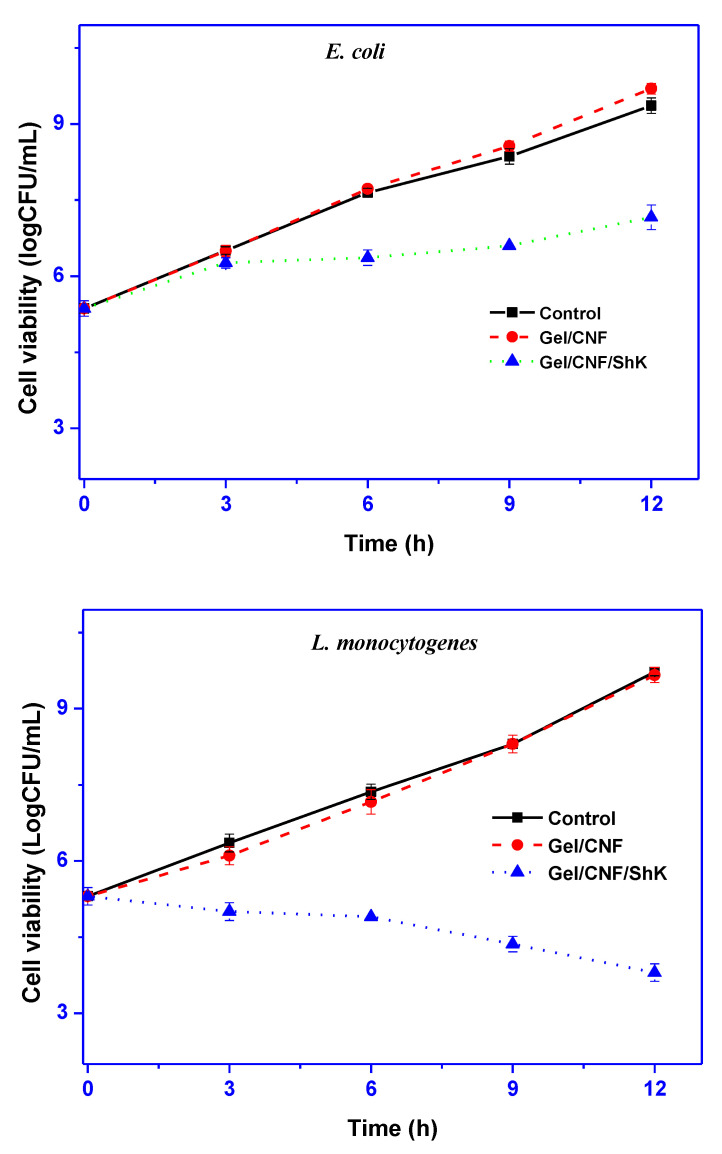
Antimicrobial activity of the film.

The effectiveness of Gel/CNF and Gel/CNF/ShK films as antibacterial agents against *L. monocytogenes* and *E. coli* was assessed using the total viable colony count method. *L. monocytogenes* and *E. coli* strains were chosen because they are representative foodborne pathogenic bacteria associated with intestinal disease and food poisoning. As shown in Figure 6, the neat Gel/CNF film did not show antibacterial activity, as expected. Depending on the type of test bacteria, the viability of bacteria drastically decreased with the addition of shikonin. The as-prepared film did not have significant antibacterial effects against *E. coli*, only marginally slowing down bacterial growth, but it did appear to have antibacterial effects against *L. monocytogenes*. The growth of *E. coli* incubated with Gel/CNF/ShK film was reduced by approximately 7 Log CFU/mL, which was approximately 3 Log CFU/mL lower than the control group. After 12 h, the Gel/CNF/ShK film showed bactericidal activity with a 6 Log CFU/mL reduction in *L. monocytogenes*.

Strong antibacterial activities against Gram-positive bacteria are widely recognized for shikonin. Shikonin’s hydrophobic properties may be responsible for its weak antibacterial effect against Gram-negative bacteria [30]. Because of the hydrophilicity of the surface, the phospholipids, proteins, and lipopolysaccharides that make up Gram-negative bacteria’s cell wall provide a permeable barrier for most hydrophobic molecules, preventing them from entering the bacterial cell [17,52]. However, due to variations in the structure of their cell walls, Gram-positive bacteria are more susceptible to shikonin since they lack an outer membrane. Gel/CNF/ShK film is anticipated to increase the shelf life of packaged foods by preventing the development or microbial contamination of such foods [53].

### 3.3. Antioxidant Activity

**Figure 7:**
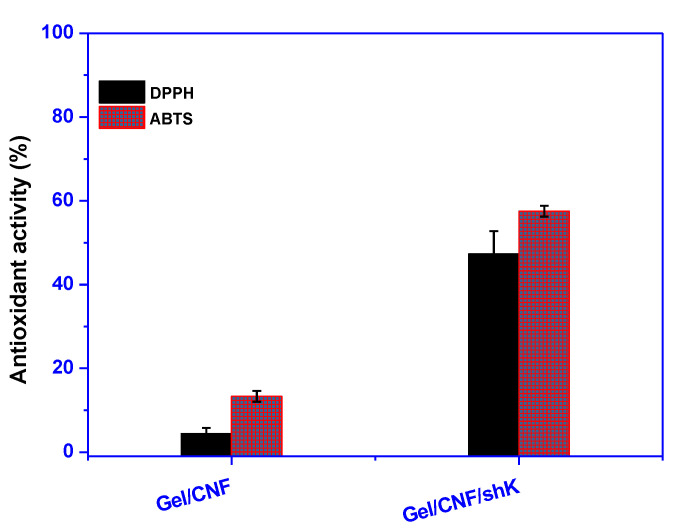
Antioxidant activity of the film.

The active antioxidants slow the lipids’ oxidation, extending the shelf life of packaged foods. Therefore, determining the film samples’ radical scavenging ability is essential [54]. The generated Gel/CNF and Gel/CNF/ShK films’ antioxidant activity is depicted in Figure 7 using the ABTS and DPPH radical scavenging assays. The results indicate that the inclusion of shikonin considerably increased the scavenging activity of produced films. Compared to Gel/CNF film, the Gel/CNF/ShK film demonstrated an ABTS antioxidant activity of ~60%, an improvement of 450%. Compared to films containing active antioxidant compounds, such as shikonin, the neat film’s 10% activity is almost negligible. Compared to the neat Gel/CNF film (5%), the DPPH antioxidant activity of shikonin-incorporated film increased to 50%, which is exceptionally high. The Gel/CNF/ShK film’s extraordinary antioxidant activity may be due to shikonin’s high levels of phenolics and flavonoids. Previous studies on the antioxidant properties of shikonin are consistent with the findings of the present investigation [13,15]. The higher antioxidant activity values in the ABTS approach compared to the DPPH method may also be partially explained by the high swelling ratio of the Gel/CNF film and enhanced interaction with free radicals. Although the methanolic DPPH solution was less likely to interact with the hydrophilic Gel/CNF surface than the ABTS method, it still exhibited less antioxidant activity [55,56]. Because of this, the Gel/CNF/ShK film is expected to limit packaged foods’ oxidative degradation and increase their shelf life.

### 3.4. Packaging Test

**Figure 8:**
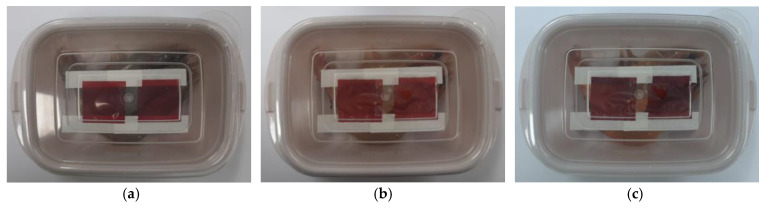
Color changes in the indicator attached to packages of shrimp, (**a**) after packaging, (**b**) after one day, and (**c**) after two days of storage at 25 °C.

To study the potentiality of the developed indicator film, the Gel/CNF/ShK film has adhered to the shrimp packaging to track how it responded to pH changes. pH is one of the major spoilage indicators of seafood, indicating the amounts of nitrogen products’ decomposition. If the pH is less than 6.5, the shrimp is considered fresh. Shrimp with a pH of less than 7 slightly deteriorates but is still edible [57]. The shrimp is severely damaged and inedible if the pH level exceeds 7. As a result, it was established that the pH value in the shrimp samples indicated the production of volatile amines (Table 3). The pH of the shrimp was 6.3 at the beginning, but it significantly increased over storage time, reaching 6.6 on the first day. The samples’ pH reading on the second day of storage was 7.1, which showed that the shrimp samples had degraded. As can be seen from Figure 8a–c, shrimp deterioration continued throughout storage, which resulted in changes to the film’s color. Dark red was initially seen as the pH indicator film’s color. The hue of the film turned red after being kept at 30 °C for one day. On the second day of storage, the pH of the shrimp increased, and the film’s reddish-blue tint was seen. These outcomes were in line with the color shift in the film’s colorimetric response to various pH levels and ammonia vapor. The color values of the film indicator altered in conformity with the data in Table 3. While the color indicator’s L, a, and b values declined over storage time, the ΔE value dramatically increased, demonstrating that the color indicator accurately predicts changes in shrimp sample quality. The current findings were consistent with those of Dong et al. (2020) and Huang et al. (2019), who also found that shikonin could be used in smart packaging to indicate the degree of meat product deterioration [13,15]. ΔE is the most important index indicating the color difference. A value of ΔE greater than 5 indicates that the changed color is visible to the naked eye, and a value greater than 12 indicates a different color [58]. The smart label in this study showed the ΔE as higher than 5 when applied for shrimp freshness monitoring during 2 days of storage.

In addition, the relationship between pH level and ΔE value was investigated to evaluate the freshness of shrimp. The ΔE of the Gel/CNF/ShK index and shrimp pH were significant and positively correlated with a correlation coefficient of 0.977. The main causes of fresh meat decomposition include microbial degradation and lipid oxidation. The meat’s pH and appearance are altered by these spoiling processes, which also cause the structural components to deteriorate [59,60]. These spoilage processes change the meat’s pH and appearance and further cause structural component degradation. This degradation releases biogenic amines and volatile organic compounds, due to microbial decarboxylation of amino acids, into the headspace, which comes in contact with the shikonin and changes its color. Therefore, the observed color shift is assumed to reflect an indirect pH measurement. The resulting color change indicates that the film can be effectively employed as a pH indicator for detecting seafood freshness.

## 4. Conclusions

The shikonin-added Gel/CNF-based film was developed for active and smart packaging applications. Shikonin was compatible with the polymer matrices, and the film showed a good pH-dependent color indicator and volatile gas sensing properties, which is ideal for food freshness monitoring. Moreover, due to the mixing of shikonin, the UV-light barrier properties, tensile strength, and hydrophobicity of the Gel/CNF film were enhanced significantly. The thermal and water vapor barrier properties were not much affected. Furthermore, due to the inclusion of shikonin, the film exhibited a promising antibacterial and antioxidant activity, which is favorable for improving the shelf-life of food. The color indicator properties of the film were used for the smart food packaging test, and the results showed it could detect and monitor shrimp’s spoilage. The dual-functioned shikonin-based film can be suitable for smart and active food packaging applications.

## Figures and Tables

**Table 1:** Surface color and light transmittance of the smart Gel/CNF films.

Films	L	a	b	ΔE	WI	T_280_ (%)	T_660_ (%)
Gel/CNF	90.9 ± 0.1 ^b^	−0.7 ± 0.1 ^a^	6.5 ± 0.1 ^a^	2.4 ± 0.1 ^a^	92.8 ± 0.1 ^b^	25.7 ± 1.3 ^b^	89.7 ± 0.4 ^b^
Gel/CNF/ShK	42.9 ± 1.6 ^a^	30.9 ± 0.6 ^b^	6.4 ± 0.4 ^a^	58.5 ± 1.7 ^b^	67.5 ± 0.7 ^a^	1.4 ± 0.3 ^a^	69.8 ± 0.8 ^a^

Any two means in the same column followed by the same letter are not significantly (*p* > 0.05) different from Duncan’s multiple range tests.

**Table 2:** Mechanical properties of films.

Films	Thickness (μm)	TS (MPa)	EB (%)	EM (GPa)
Gel/CNF	67.2 ± 5.3 ^a^	69.6 ± 7.2 ^a^	4.3 ± 0.6 ^a^	3.2 ± 0.9 ^a^
Gel/CNF/ShK	67.3 ± 4.0 ^a^	83.0 ± 12.8 ^b^	5.2 ± 1.5 ^a^	4.2 ± 0.3 ^b^

Any two means in the same column followed by the same letter are not significantly (*p* > 0.05) different from Duncan’s multiple range tests.

**Table 3:** Changes in the color values of the indicator and the pH of shrimp sample papers during storage. Correlation of ΔE of the indicator with pH during storage.

Time (h)	L	a	b	ΔE	pH	R
0 h	47.4 ± 2.1	30.5 ± 0.3	12.5 ± 0.2	-	6.3	0.977
24 h	45.3 ± 1.4	27.6 ± 1.6	10.4 ± 1.1	1.2 ± 0.3	6.6	
48 h	30.8 ± 0.4	4.6 ± 0.2	0.7 ± 0.3	6.7 ± 0.7	7.1	

## Data Availability

Not applicable.

## Supplementary Materials

The following supporting information can be downloaded at: https://www.mdpi.com/article/10.3390/ma15196615/s1, Figure S1: (a) Color of shikonin solution and (b) UV-vis spectra of shikonin solution at various pH. Table S1: Color response of films at various pH values, Table S2: Water vapor permeability, water contact angle, moisture content, water solubility, and swelling ratio of films. References [61,62,63,64,65] are cited in the Supplementary Materials Files.
