# Supplementary material for: Shikonin Functionalized Packaging Film for Monitoring the Freshness of Shrimp
Source: Materials (Basel). 2022 Sep 23;15(19):6615. doi: 10.3390/ma15196615 (PMC9572350; doi:10.3390/ma15196615)
Supplement: Supplementary file 1 [file materials-15-06615-s001.zip › materials-1918481-supplementary.pdf]

materials

Supplementary Files

Shikonin Functionalized Packaging Film for Monitoring the Freshness of Shrimp

Swarup Roy [1,2,*,†], Parya Ezati [1,†], Deblina Biswas [2] and Jong-Whan Rhim [1,*]

1 Department of Food and Nutrition, BioNanocomposite Research Institute, Kyung Hee University, 26 Kyungheedae-ro, Dongdaemun-gu, Seoul 02447, Korea
2 School of Bioengineering and Food Technology, Shoolini University, Bajhol 173229, India
* Correspondence: swaruproy2013@gmail.com (S.R.); jwrhim@khu.ac.ke (J.-W.R.)
† These authors contributed equally to this work.

Materials and Methods

Characterization and properties of the film

Color response efficacy, surface color, and transmittance

The color variation of shikonin and shikonin-added indicator film was checked using pH 2-12 buffer solutions [1]. The Hunter color (L, a, and b) and total color difference (ΔE) of the film sample were also measured using a Chroma meter (Konica Minolta, CR-400, Tokyo, Japan) using a white standard plate as a background. The total color difference (ΔE) and the whiteness index (WI) were calculated using the following equation.

$$\Delta E = \sqrt{(\Delta L)^2 + (\Delta a)^2 + (\Delta b)^2} \quad (S1)$$

$$WI = 100- [(100-L)+(a)^2+(b)^2]^{0.5} \quad (S2)$$

where ΔL, Δa, and Δb were differences between each color value of the control film and the test film specimen.

The volatile gas sensitivity of the intelligent film to acid and base was checked [1]. The dry film sample was hung over an acetic acid or an ammonia solution for 10 min, then detached and captured digital color images.

The UV-barrier and transparency properties of the films specimen were also assessed by determining the percent transmittance of the film sample (5 cm × 5 cm) at 280 nm (T_{280}) and 660 nm (T_{660}), respectively, in a UV-vis spectrophotometer (Mecasys Optizen POP Series UV/Vis, Seoul, Korea) [2].

Morphology and FTIR

The films' surface and cross-section topology was inspected using the FESEM (FE-SEM, SU 8010, Hitachi Co., Ltd., Matsuda, Japan). All the film samples were sputter-coated with platinum for 2 min before the measurement. The FTIR spectra of all the films were noted in the ATR mode using an FTIR spectrometer (TENSOR 37 Spectrophotometer with OPUS 6.0 software, Billerica, MA, USA) wavenumber ranging from 4000-650 cm^{-1}.

Water vapor permeability (WVP) and water contact angle (WCA)

The water vapor permeability of all the films was measured gravimetrically using a WVP cup by following the ASTM E96-95 standard method [3]. The WVP cup was first filled with a prescribed amount of water, then covered with films (5 cm × 5 cm), sealed, and kept in the controlled environmental chamber (25 °C and 50% RH). After equilibration, the WVP cup's weight was checked at a pre-decided time interval, and the weight loss was determined. The WVTR ($g/m^2.s$) was determined from the slope (linear) of the steady-state portion of weight loss of the cup versus the time curve. Then, the WVP of the films was calculated in $g.m/m^2.Pa.s$ using the following equation:

$$WVP = (WVTR \times L)/\Delta p \quad (S3)$$

where L was the thickness of the film (m), and Δp was the water vapor partial pressure difference (Pa) across the film [2].

The film's surface wettability was evaluated by measuring the water contact angle of the film surface using a WCA analyzer (Phoneix 150, Surface Electro Optics Co., Ltd., Kunpo, Korea). The film sample (3 cm × 10 cm) was fixed on the film holder, and a drop of water (~10 μL) was added to the film's surface and immediately measured the WCA [2].

Moisture content (MC), swelling ratio (SR), and water solubility (WS)

Moisture content (MC), swelling ratio (SR), and water solubility (WS) of the films was determined using the previously reported methods [4]. The films' MC was determined by measuring the weight change of the film after drying at 105 °C for 24 h. The percentage of MC content of the films was calculated using the following equation:

$$\mathrm{MC}\ (\%) = \frac{W_i - W_f}{W_i} \times 100 \qquad \text{(S4)}$$

where W_i and W_f refer to the initial and final mass of the film samples, respectively.

A pre-weighed film sample was put in a beaker containing 20 mL DI water, taken out from the water after one h, wiped out the surface water using blotting paper, and then weighed [5]. The SR of the films was calculated in triplicate using the following equation:

$$\mathrm{SR}\ (\%) = \frac{W_f - W_i}{W_i} \times 100 \qquad \text{(S5)}$$

where W_i and W_f refer to the initial and final weight of the film samples, respectively.

The pre-dried film sample was put in a beaker that contained 30 mL of DI water, and the beaker was covered and kept for 24 h at room temperature with gentle agitation [4]. The film specimen was removed, dried in a hot air oven at 105 °C for 24 h, and weighed. The WS of the film sample was calculated using the following equation:

$$\mathrm{WS}\ (\%) = \frac{W_i - W_f}{W_i} \times 100 \qquad \text{(S6)}$$

Mechanical and thermal properties

Film thickness was measured using an electronic digital micrometer (Digimatic Micrometer, QuantuMike IP 65, Mitutoyo, Japan) with an accuracy of 1 µm. The film's mechanical properties were determined following the standard method (ASTM D 882-88) using an Instron Universal Testing Machine (Model 5565, Instron Engineering Corporation, Canton, MA, USA). Rectangular strip (2.54 cm × 15 cm) film samples were used for measurement. The Instron machine was operated in tensile mode with an initial grip separation of 50 mm and a 50 mm/min crosshead speed [2].

The film's thermal stability was evaluated using a thermogravimetric analyzer (Hi-Res TGA 2950, TA Instrument, New Castle, DE, USA). For this, ~10 mg of film sample was taken in a standard aluminum pan and scanned in a temperature range of 30-600 °C under nitrogen flow (50 mL/min) [2].

Antibacterial and antioxidant activity

The films' antibacterial activity was calculated using a total viable colony count (TVCC) method [6]. The food-borne pathogenic bacteria, *L. monocytogenes* and *E. coli*, were used in this test. The test bacteria were inoculated in the brain heart infusion (BHI) and tryptic soy broth (TSB), respectively, cultured overnight at 37 °C with gentle agitation for 16 h. The inoculum was then suitably diluted, and ~100 µL of the diluted inoculum was aseptically transferred to the broth of TSB and BHI containing ~150 mg of the film samples incubated at 37 °C with gentle agitation. The sample was taken out and plated on agar plates at regular intervals (3, 6, 9, and 12 h) after suitable dilution to evaluate the TVCC. The antibacterial test was also carried out using a culture medium without film, and a control film served as negative control and positive control, respectively.

Antioxidant activities of the films were evaluated using DPPH and ABTS radical scavenging methods [7]. The assay solution was prepared as per the earlier published recipe. The film samples were mixed with DPPH and ABTS assay solution in the dark for 30 min at room temperature in the dark and measured the absorbance at 517 nm and 734 nm, respectively, using a UV-vis spectrophotometer. The antioxidant activity of all the tested films was calculated using the following equation.

$$\text{Free radical scavenging activity}\ (\%) = \frac{A_c - A_s}{A_c} \times 100 \qquad \text{(S7)}$$

where A_c and A_s were the absorbance of DPPH/ABTS of the control and test film, respectively.

Application of indicators for shrimp packaging

The pH-sensitive indicator was placed on the inner side of the packaging lid, and 100 g of fresh samples were packed into the boxes [8]. The shrimp samples were then kept at 25 °C for 48 hours. The technique described in the color property section was also used to measure the color of the attached pH-sensitive indicator in three replicates. For the pH of the shrimp sample, a 10 g sample was homogenized with 90 mL of distilled water, and the pH of the resulting slurry was measured using a digital pH meter (Thermo Scientific, Indonesia). The correlation between pH and ΔE was determined by Pearson's correlation method.

Statistical analysis

All the tests were performed in triplicate, and the mean±standard deviation value was reported. One-way analysis of variance (ANOVA) was done, and the significance of each mean property value was evaluated ($p < 0.05$) by Duncan's multiple range test using the SPSS statistical analysis (SPSS Inc., Chicago, IL, USA).

Results

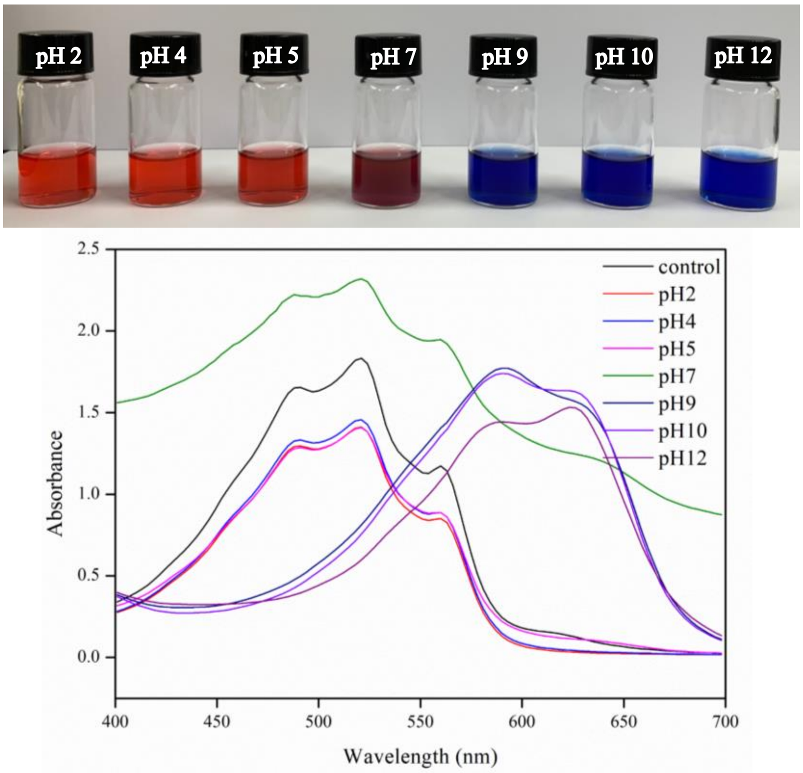

Figure S1. (a) Color of shikonin solution and (b) UV-vis spectra of shikonin solution at various pH.

Table S1. Color response of films at various pH values.

	Control film	pH=2	pH=4	pH=5	pH=7	pH=9	pH=10	pH=12
L	41.2±0.2[a]	46.7±1.9[a]	36.3±2.3[c]	43.8±0.7[c]	35.4±0.4[c]	47.6±1.4[c]	31.3±0.3[c]	33.8±1.5[c]
a	30.6±0.1[a]	21.2±0.2[a]	26.1±1.3[b]	26.4±0.2[b]	26.0±0.2[b]	21.4±1.2[b]	21.1±0.6[b]	11.4±0.3[b]
b	6.1±0.0[b]	7.1±0.1[b]	6.8±0.3[a]	6.6±0.0[a]	5.7±0.1[a]	1.8±0.7[a]	-3.5±0.2[a]	-10.3±0.3[a]
ΔE	59.6±0.2[b]	50.4±1.6[b]	61.8±1.5[a]	55.3±0.5[a]	62.5±0.3[a]	49.6±1.6[a]	65.0±0.1[a]	59.6±0.2[a]

Any two means in the same column followed by the same letter are not significantly ($p > 0.05$) different from Duncan's multiple range tests.

Table S2. Water vapor permeability, water contact angle, moisture content, water solubility, and swelling ratio of films.

Films	WVP ($\times 10^{-9}$ g.m/m^2.Pa.s)	WCA (deg.)	MC (%)	WS (%)	SR (%)
Gel/CNF	0.86±0.1[a]	60.3± 1.0[a]	6.7±0.9[a]	47.8±4.6[a]	538.1±9.6[b]
Gel/CNF/ShK	0.89±0.1[a]	66.5± 3.4[b]	5.6±0.6[a]	45.2±6.1[a]	277.0±10.4[a]

Any two means in the same column followed by the same letter are not significantly ($p > 0.05$) different from Duncan's multiple range tests.

References

1. Yun, D.; Cai, H.; Liu, Y.; Xiao, L.; Song, J.; Liu, J. Development Of Active And Intelligent Films Based On Cassava Starch And Chinese Bayberry (Myrica Rubra Sieb. Et Zucc.) Anthocyanins. *RSC Adv.* **2019**, *9*, 30905–30916. https://doi.org/10.1039/C9RA06628D
2. Roy, S.; Rhim, J.-W.; Jaiswal, L. Bioactive Agar-Based Functional Composite Film Incorporated with Copper Sulfide Nanoparticles. *Food Hydrocoll.* **2019**, *93*, 156–166. https://doi.org/10.1016/J.FOODHYD.2019.02.034.
3. Rhim, J.-W. Physical-Mechanical Properties of Agar/κ-Carrageenan Blend Film and Derived Clay Nanocomposite Film. *J. Food Sci.* **2012**, *77*, N66–N73. https://doi.org/10.1111/j.1750-3841.2012.02988.x.

4. Roy, S.; Kim, H.C.; Kim, J.W.; Zhai, L.; Zhu, Q.Y.; Kim, J. Incorporation of Melanin Nanoparticles Improves UV-Shielding, Mechanical and Antioxidant Properties of Cellulose Nanofiber Based Nanocomposite Films. *Mater. Today Commun.* **2020**, *24*, 100984. https://doi.org/10.1016/j.mtcomm.2020.100984.
5. Huang, S.; Xiong, Y.; Zou, Y.; Dong, Q.; Ding, F.; Liu, X.; Li, H. A Novel Colorimetric Indicator Based on Agar Incorporated with Arnebia Euchroma Root Extracts for Monitoring Fish Freshness. *Food Hydrocoll.* **2019**, *90*, 198–205. https://doi.org/10.1016/J.FOODHYD.2018.12.009.
6. Ferreira, A.S.; Nunes, C.; Castro, A.; Ferreira, P.; Coimbra, M.A. Influence Of Grape Pomace Extract Incorporation On Chitosan Films Properties. Carbohydrate. *Polymers*. **2014**, *113*, 490–499. https://doi.org/10.1016/j.carbpol.2014.07.032
7. Oun, A.A.; Bae, A.Y.; Shin, G.H.; Park, M.K.; Kim, J.T. Comparative Study Of Oregano Essential Oil Encapsulated In Halloysite Nanotubes And Diatomaceous Earth As Antimicrobial And Antioxidant Composites. *Appl. Clay Sci.* **2022**, *224*, 106522. https://doi.org/10.1016/j.clay.2022.106522
8. Ghorbani, M.; Divsalar, E.; Molaei, R.; Ezati, P.; Moradi, M.; Tajik, H.; Abbaszadeh, M. A Halochromic Indicator Based on Polylactic Acid and Anthocyanins for Visual Freshness Monitoring of Minced Meat, Chicken Fillet, Shrimp, and Fish Roe. *Innov. Food Sci. Emerg. Technol.* **2021**, *74*, 102864. https://doi.org/10.1016/J.IFSET.2021.102864.
